# Supplementary material for: Facile Fabrication of a Novel PZT@PPy Aerogel/Epoxy Resin Composite with Improved Damping Property
Source: Polymers (Basel). 2019 Jun 3;11(6):977. doi: 10.3390/polym11060977 (PMC6630557; doi:10.3390/polym11060977)
Supplement: Supplementary file 1 [file polymers-11-00977-s001.pdf]

Supporting Information

Facile fabrication of a novel PZT@PPy aerogel/epoxy resin composite with improved damping property

Chunmei Zhang[a], Yuchao Li[a,*], Yanhu Zhan[a], Qian Xie[a]

[a]School of Materials Science and Engineering, Liaocheng University, Liaocheng, 252059, China

*Corresponding author: Yuchao Li (email: liyuchao@lcu.edu.cn)

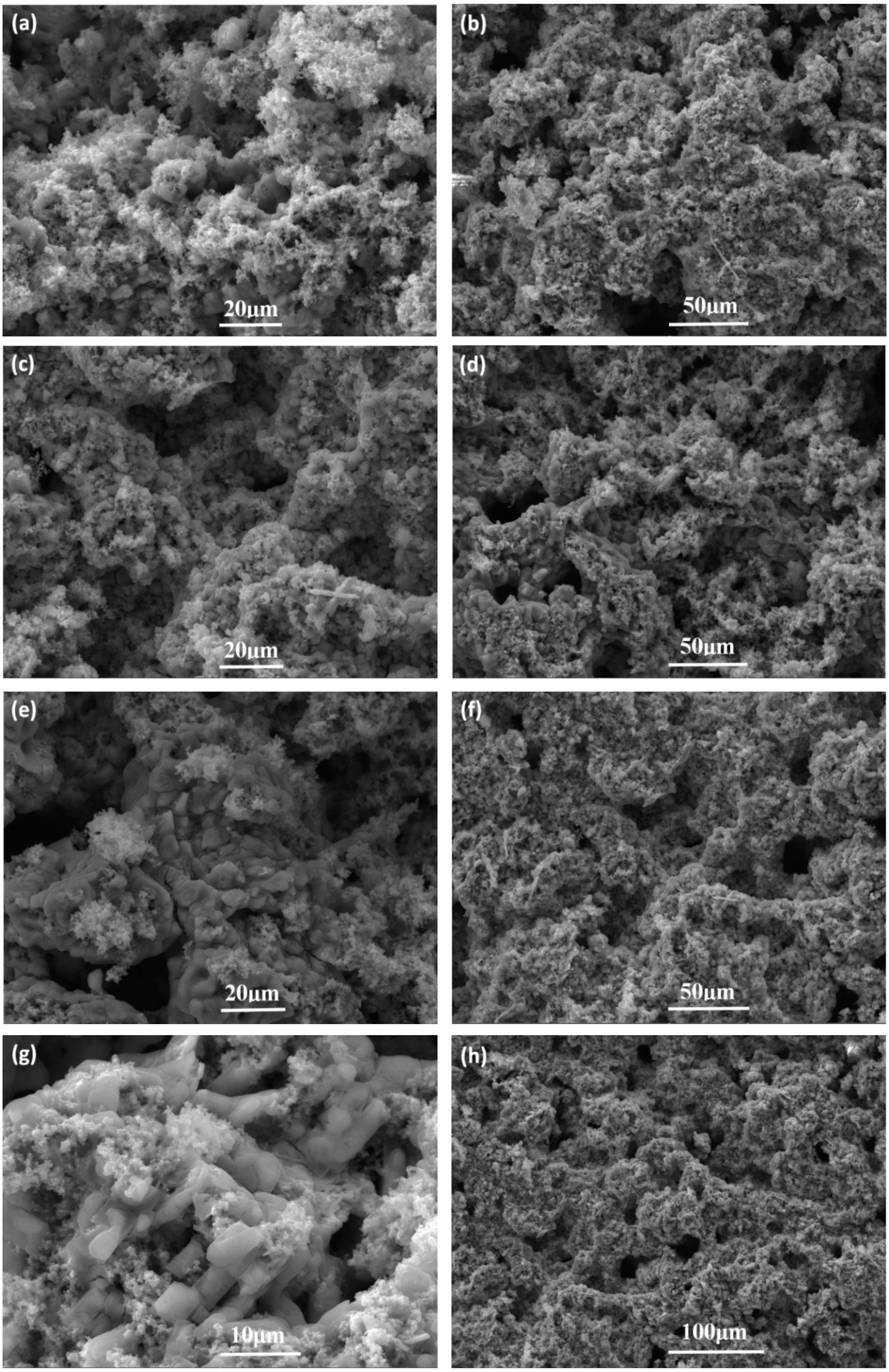

Figure S1 The SEM images of the PPAs with different content of PZT ceramics: (a) and (b) 25 wt%, (c) and (d) 50 wt%, (e) and (f) 100 wt%, and (g) and (h) 75 wt%

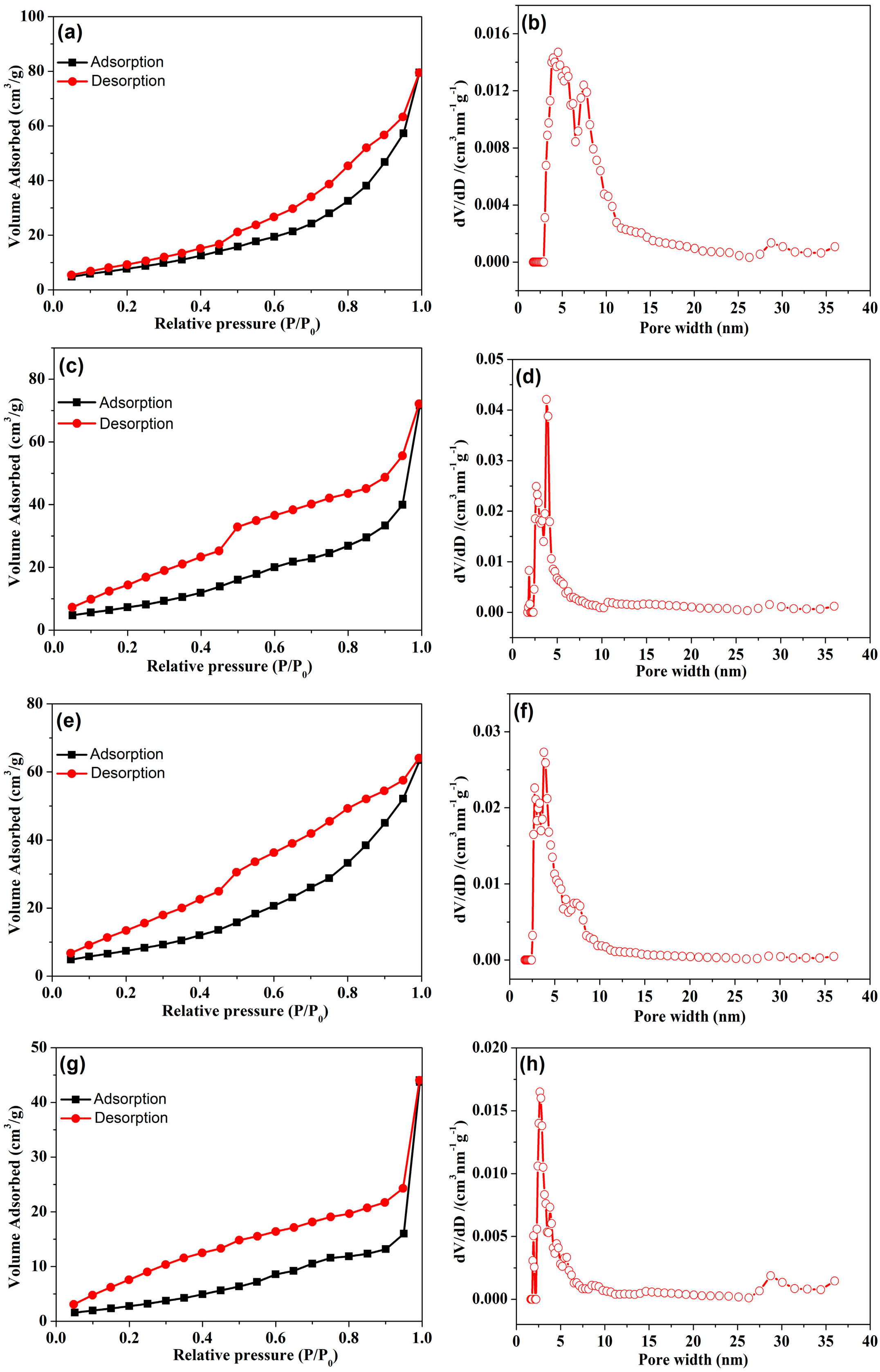

Figure S2 The nitrogen adsorption and desorption isotherms and the pore size distribution plots of PPAs with different content of PZT ceramics: (a) and (b) 0 wt%, (c) and (d) 25 wt%, (e) and (f) 50 wt%, and (g) and (h) 100 wt%

Table S1 The results of the nitrogen adsorption and desorption measurements of PPAs with different content of PZT ceramics

Sample	BET surface area (m^2/g)	DFT adsorption average pore diameter (nm)	DFT adsorption cumulative pore volume (cm^3/g)
PPA-0	32.100	4.543	0.102
PPA-25	29.965	3.794	0.090
PPA-50	29.961	3.794	0.088
PPA-75	28.314	3.794	0.063
PPA-100	12.663	2.647	0.045

Preparation of samples for different measurements

The PPAs were cut into pieces, and the aerogel blocks were used directly for BET and SEM measurement. After saturating the PPAs with epoxy resin, the PPAEs were obtained. The PPAE composite were cut into rectangular specimens of 10 × 10 × 2 mm for SEM measurement. The PPAE composite were cut into rectangular specimens of 60 × 60 × 2 mm for volume resistivity measurement using a ZC-36 high resistance meter. The PPAE composite were cut into rectangular specimens of 30 × 8 × 2 mm for Dynamic mechanical measurements by Perkin-Elmer DMA 8000.
